# The association between non-high-density lipoprotein cholesterol to high-density lipoprotein cholesterol ratio (NHHR) and prevalence of periodontitis among US adults: a cross-sectional NHANES study

**DOI:** 10.1038/s41598-024-56276-y

**Published:** 2024-03-06

**Authors:** Kegui Hou, Wenpeng Song, Jun He, Zhaofeng Ma

**Affiliations:** 1 Beijing Shunyi District Hospital, Beijing, 101300 China; 2 Beijing Tiantan Hospital, Capital Medical University, Beijing, 100070 China

**Keywords:** The non-high-density lipoprotein cholesterol to high-density lipoprotein cholesterol ratio, Periodontitis, NHANES, A cross-sectional study, Computational biology and bioinformatics, Risk factors

## Abstract

The non-high-density lipoprotein cholesterol to high-density lipoprotein cholesterol ratio (NHHR) is a recently developed lipid parameter, but there is currently a lack of research exploring its relationship with periodontitis. This study aims to identify the potential association between NHHR and periodontitis. The association between NHHR and periodontitis were examined through univariate and multivariate weighted logistic regression utilizing the National Health and Nutrition Examination Survey data from 2009 to 2014. The participants were grouped based on the type of periodontitis. This study included a total of 9023 participants, with 1947 individuals having no periodontitis, and an additional 7076 individuals suffering from periodontitis. Patients in periodontitis group demonstrated a statistically significant elevation in NHHR values 2.82 (2.05–3.80) compared to those in no periodontitis group (*p* < 0.001). Logistic regression analysis of variables demonstrated a positive association between NHHR and periodontitis [1.07 (1.02, 1.12) *p* = 0.0067]. The study revealed a positive association between NHHR and an elevated prevalence of periodontitis development. For each unit increase in NHHR, there is a 7% increase in the prevalence of periodontitis. Further investigations into NHHR may enhance our understanding of preventing and treating periodontitis. However, additional studies are required to validate these findings.

## Introduction

Periodontitis is a chronic, multifactorial inflammatory disease associated with the accumulation of dental plaque, characterized by progressive destruction of the supporting structures of teeth, including the periodontal ligament and alveolar bone^1–3^. The research findings from 2017 indicate that approximately 796 million people worldwide are affected by periodontitis^4^. While causing physical and mental distress to patients, this has also emerged as a global public health challenge^5,6^. In the United States, 42.2% of adults (≥ 30 years of age) have periodontitis, and within that group, 7.8% have severe periodontitis^7^.

In recent years, the gradual advancement in the etiology and epidemiology of periodontitis has provided evidence for the association between periodontitis and systemic diseases, including diabetes, cardiovascular diseases, metabolic disorders, respiratory diseases, rheumatoid arthritis, certain cancers, and cognitive disorders^8,9^. Certain studies have highlighted that the onset of periodontitis triggers the release of inflammatory agents into the bloodstream, leading to persistent low-level inflammation throughout the body, thereby affecting systemic health^10–12^.

Due to the lack of easily identifiable symptoms in the early stages, periodontitis is often overlooked until it progresses to a potentially irreversible stage, where loosening and loss of teeth may occur^2^. There is an urgent need to identify new biological markers, preferably obtainable through blood tests, for assessing individuals' susceptibility to periodontitis. Blood tests are common medical tests and are part of a routine physical examination^13,14^. This approach is crucial to enhance current prevention and treatment strategies of periodontitis.

The association between common lipoproteins and periodontitis has been widely discussed in previous reports. The high-density lipoprotein cholesterol (HDL-C), a lipid with anti-inflammatory and antioxidant properties, can modulate innate and adaptive immune responses^15^. It has been suggested that periodontal pathogenic bacteria, along with the resulting metabolites and pro-inflammatory cytokines from periodontal infection, may contribute to HDL metabolism impairment and peroxidation^16,17^. While numerous studies have reported a significant decrease in HDL levels among patients with periodontitis, it is worth noting that some research has not found a statistically significant difference in HDL between patients with periodontitis and those without^18–21^. Developing a more reliable blood lipid parameter as a predictive indicator for periodontitis and harnessing its potential utility in periodontal risk assessment and preventive decision-making could benefit a large population of periodontitis patients.

The non-high-density lipoprotein (NHDL) cholesterol to HDL-C ratio (NHHR) is an emerging comprehensive indicator of atherosclerotic lipid^22^. Previous studies have indicated that, compared to standard blood lipid parameters, NHHR demonstrates superior predictive and diagnostic efficacy in assessing the risks of atherosclerosis, non-alcoholic fatty liver disease, chronic kidney disease, insulin resistance, and metabolic syndrome^23–26^. In addition, recent research has also reported the association and predictive value of NHHR with various diseases such as abdominal aortic aneurysm, diabetes, and depression^27–29^.

To the best of our knowledge, there are currently no reports on the relationship between NHHR and periodontitis. We hypothesize that there may be a correlation between NHHR and periodontitis. Managing and controlling NHHR could potentially contribute to the prevention and management of periodontal disease. In this regard, we conducted a cross-sectional study based on the NHANES 2009–2014 dataset to explore the association between NHHR and the prevalence of periodontitis in US adults.

## Materials and methods

### Study population

The data utilized in this study are sourced from the NHANES 2009–2014, a research program aimed at evaluating the wellbeing and nutritional condition of adults and children in the United States. The survey comprises demographic, socio-economic, dietary, and health-related inquiries. The screening segment encompasses medical, dental, and physiological measurements alongside laboratory tests conducted by trained medical personnel. The survey results will establish the occurrence rates of significant diseases and disease-causing prevalences. Further details are available online (https://www.cdc.gov/nchs/nhanes/index.htm).

**Figure 1:**
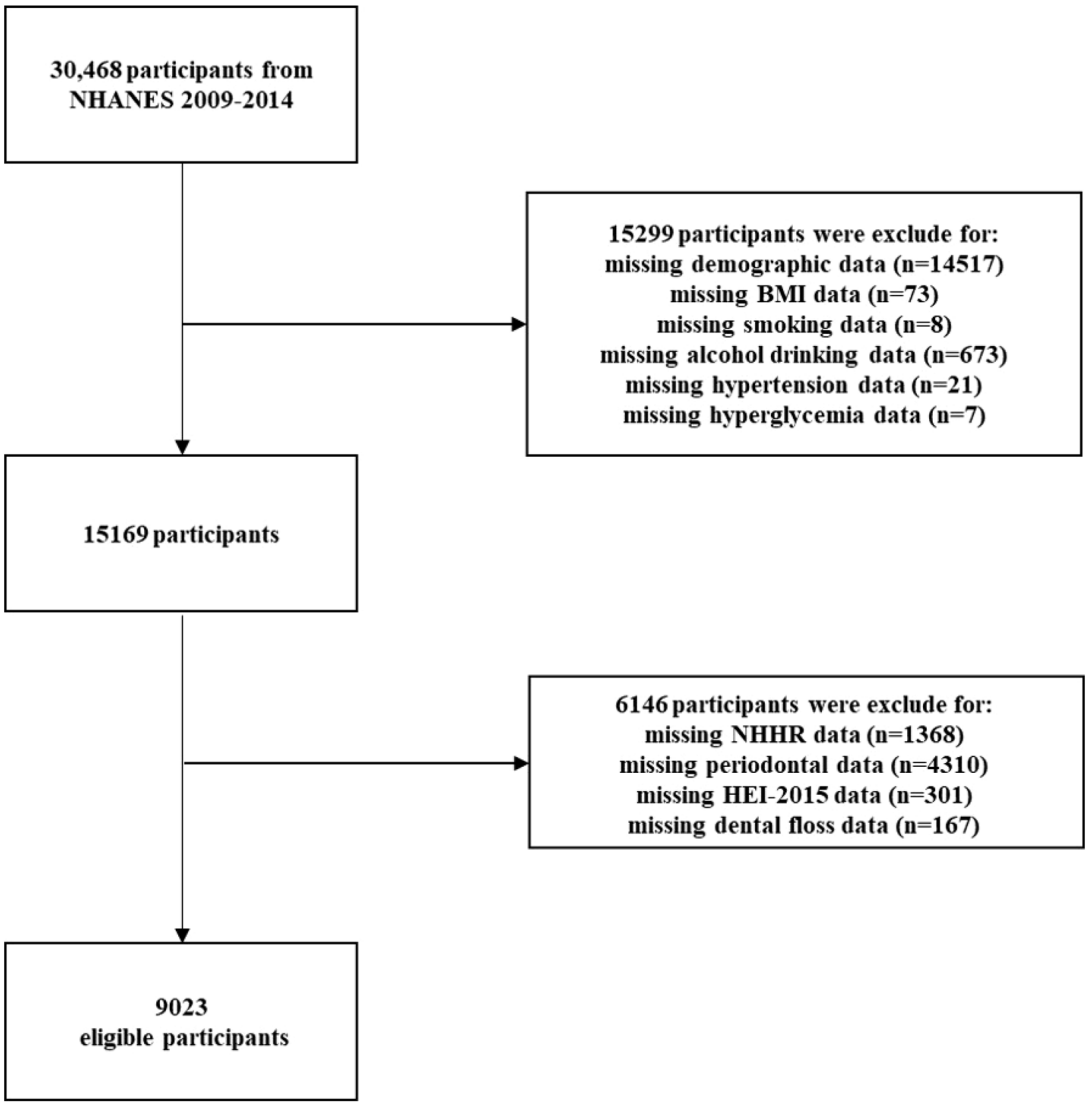
Flowchart of study participants selection.

A total of 30,468 individuals participated in NHANES between 2009 and 2014. However, after applying exclusion criteria, the sample size for this study was reduced to 9,023 participants. The exclusion criteria included incomplete demographics data, missing data on covariates (such as smoking status, BMI, alcohol use, the use of dental floss, the number of tooth loss, diet level, history of hypertension, and hyperglycemia), and lack of periodontal examination or NHHR-related data (total cholesterol and high-density lipoprotein data). Figure 1 shows the screening process flowchart.

### The calculation of NHHR

The data source for the NHHR calculations is derived from laboratory data in NHANES called ‘HDL.Doc’ which provides HDL data and ‘TCHOL.Doc’, which provides total cholesterol data. The NHHR data is obtained using the formula for total cholesterol minus HDL, then divided by HDL.

### Assessment of periodontitis

The NHANES examination data includes a file named “OHXPER.Doc” that encompasses data on periodontal examinations involving clinical attachment loss (CAL) and probing depth (PD). This data classified participants as periodontitis or not based on criteria provided by the Centers for Disease Control and Prevention and the American Academy of Periodontology (CDC/AAP)^30^.

Severe periodontitis was determined by the presence of at least two interproximal regions exhibiting a clinical attachment level (CAL) of no less than 6 mm and not located on the same tooth, alongside at least one interproximal site exhibiting a probing depth (PD) of no less than 5 mm. Moderate periodontitis was defined as the identification of two or more interproximal regions with probing pocket depths that are greater than or equal to 5 mm and not situated on the same tooth, or two or more interproximal areas with clinical attachment levels that are greater than or equal to 4 mm and not found on the same tooth. Participants diagnosed with mild periodontitis must exhibit at least two interproximal sites with a clinical attachment loss of at least 3 mm, and at least two interproximal sites with a probing depth of at least 4 mm (not on the same tooth) or one site with a probing depth of at least 5 mm. In this study, we grouped mild, moderate, and severe periodontitis into one category (having periodontitis), and no periodontitis in another^31,32^.

### Treatment of covariates

To explore the relationship between NHHR and periodontitis, several covariates were selected for adjustment, encompassing demographic data, lifestyle habits, and health status.

Demographic data comprised age, gender, race, education level, marital status, and income. Lifestyle behaviors encompassed the use of alcohol, smoking status, the use of dental floss and dietary levels. “Alcohol use” was judged by “Had at least 12 alcohol drinks/1 year?”. Smoking status was determined by “Have you smoked at least 100 cigarettes in your entire life?”^31^. The frequency of flossing was determined based on the number of days reported in the questionnaire. Participants who reported not flossing at all during the week were labeled as “No”. Dietary levels are determined by the HEI-2015 score, which considers 13 dietary components. Each component is scored and added together to obtain the final result. Higher scores indicate higher dietary levels^33^. Health status involved measuring BMI, the number of tooth loss, hypertension, and hyperglycemia, which can be obtained directly from the questionnaires and measurement reports.

### Statistical analyze

All statistical analyses were conducted using R (version 4.2), SPSS (version 26.0) and Empowerstats (version 5.0), and the NHANES guidelines were used to weight all data. Natural cubic spline was used to identify the relationship of NHHR with periodontitis. Then, we performed logistic regression analyses to examine the correlation between NHHR and periodontitis^34^, followed by subgroup analyses to investigate potential differences in the correlations regarding gender, age, race, income, education, and marital status etc.

### Ethics approval and consent to participate

The NHANES are public database. The patients involved in the database received ethical approval. Users can download relevant data for free for research and publication purposes.

## Results

### Characteristics of participants

The characteristics of participants are shown in Table 1. The study includes 9023 individuals with an average age of 52.00 years. Among them, 78.42% were diagnosed with periodontitis. The NHHR of this group was 2.82 (2.05–3.80) which was statistically significant when compared to the NHHR of none periodontitis patients [2.67 (1.96–3.61), (*P* < 0.001)]. Significant differences in characteristics were observed between participants with none periodontitis and periodontitis with regards to age, gender, race, education level, marital status, ratio of family income to poverty, HEI-2015, the number of tooth loss and total cholesterol.

**Table 1:** Characteristics of participants.

	Total (N = 9023)	None periodontitis (N = 1947)	Periodontitis (N = 7076)	*P* value
Age (years)	52.00 (41.00–64.00)	49.00 (37.00–66.00)	53.00 (42.00–64.00)	< 0.001
Gender				< 0.001
Male	4569 (50.64%)	770 (39.55%)		3799 (53.69%)
Female	4454 (49.36%)	1177 (60.45%)	3277 (46.31%)	
Race				< 0.001
Mexican American	1162 (12.88%)	133 (6.83%)	1029 (14.54%)	
Other Hispanic	831 (9.21%)	157 (8.06%)	674 (9.53%)	
Non-Hispanic white	4292 (47.57%)	1126 (57.83%)	3166 (44.74%)	
Non-Hispanic black	1827 (20.25%)	360 (18.49%)	1467 (20.73%)	
Other race	911 (10.10%)	171 (8.78%)	740 (10.46%)	
Education level				0.030
Less than 9th grade	810 (8.98%)	168 (8.63%)	642 (9.07%)	
9–11th grade	1249 (13.84%)	275 (14.12%)	974 (13.76%)	
High school grad	2022 (22.41%)	398 (20.44%)	1624 (22.95%)	
Some college or AA degree	2570 (28.48%)	546 (28.04%)	2024 (28.60%)	
College graduate or above	2372 (26.29%)	560 (28.76%)	1812 (25.61%)	
Marry status				< 0.001
Married	5206 (57.70%)	1124 (57.73%)		4082 (57.69%)
Widowed	739 (8.19%)	207 (10.63%)	532 (7.52%)	
Divorced	1207 (13.38%)	263 (13.51%)	944 (13.34%)	
Separated	328 (3.64%)	53 (2.72%)	275 (3.89%)	
Never married	978 (10.84%)	200 (10.27%)	778 (10.99%)	
Living with partner	565 (6.26%)	100 (5.14%)	465 (6.57%)	
Ratio of family income to poverty				0.005
< 1.3	2715 (30.09%)	579 (29.74%)		2136 (30.19%)
1.3–3.5	3241 (35.92%)	650 (33.38%)	2591 (36.62%)	
> 3.5	3067 (33.99%)	718 (36.88%)	2349 (33.20%)	
BMI	28.38 (24.80–32.80)	28.09 (24.50–32.78)	28.40 (24.90–32.80)	0.063
Smoking status				0.842
Yes	4226 (46.84%)	908 (46.64%)	3318 (46.89%)	
No	4797 (53.16%)	1039 (53.36%)	3758 (53.11%)	
Alcohol use				0.476
Yes	6656 (73.77%)	1424 (73.14%)	5232 (73.94%)	
No	2367 (26.23%)	523 (26.86%)	1844 (26.06%)	
Hypertension				0.167
Yes	3603 (39.93%)	751 (38.57%)	2852 (40.31%)	
No	5420 (60.07%)	1196 (61.43%)	4224 (59.69%)	
Hyperglycemia				0.789
Yes	1237 (13.71%)	313 (16.08%)	1183 (16.72%)	
No	7786 (86.29%)	1634 (83.92%)	5893 (83.28%)	
HEI-2015	51.61 (42.06–61.58)	51.12 (41.45–60.39)	51.77 (42.21–61.94)	0.007
Tooth loss	3.00 (0.00–9.00)	2.00 (0.00–4.00)	4.00 (1.00–10.00)	< 0.001
Dental floss				< 0.001
Yes	5803 (64.31%)	942 (48.38%)	2278 (32.19%)	
No	3220 (35.69%)	1005 (51.62%)	4798 (67.81%)	
HDL-C(mg/dL)	50.00 (41.00–61.00)	50.00 (41.00–62.00)	50.00 (41.00–61.00)	0.101
Total Cholesterol (mg/dL)	194.00 (168.00–222.00)	190.00 (165.00–218.00)	195.00 (169.00–223.00)	< 0.001
NHHR	2.79 (2.03–3.76)	2.67 (1.96–3.61)	2.82 (2.05–3.80)	< 0.001

*NHHR* non-high-density lipoprotein cholesterol to high-density lipoprotein cholesterol ratio, *HEI-2015* The Healthy Eating Index 2015, *BMI* body mass index, *HDL-C* high density lipoprotein cholesterol.

### Association between NHHR and periodontitis

**Figure 2:**
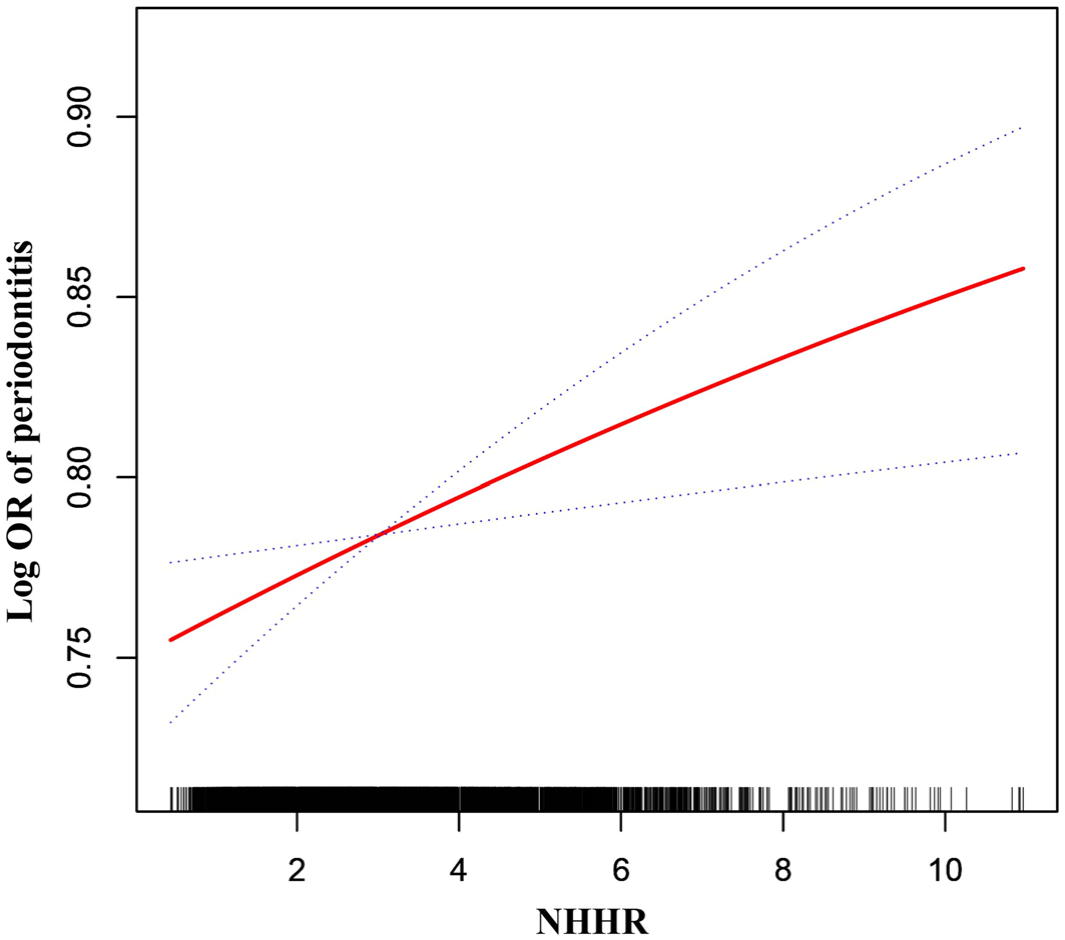
The association between NHHR and periodontitis.

The association between NHHR and periodontitis were displayed in Table 2. Following multiple adjustments, NHHR was found to be positively correlated with periodontitis [1.07 (1.02, 1.12), *p* = 0.0067] in the model 3, while the smoothed curve fitting confirmed this positive correlation (Fig. 2). The goodness of fit results in Table 2 showed that Model 3 (R^2^ = 17.5%) has a better predictive ability than the other models.

**Table 2:** Association between NHHR and periodontitis among participants in NHANES 2009–2014.

	OR	*P* value	*R*^2^ (%)*
Model 1	1.08 (1.04, 1.12)	0.0001	0.2%
Model 2	1.04 (1.00, 1.08)	0.0705	3.4%
Model 3	1.07 (1.02, 1.12)	0.0067	17.9%

Model 1 did not adjust for any potential confounders;

Model 2 adjusted for: gender, age, race;

Model 3 adjusted for: gender, age, race, education level, pir, smoking status, hypertension, hyperglycemia, HDL-c, total cholesterol, BMI, marry status, Alcohol use, HEI-2015 score, dental floss use status, tooth loss number.

*Goodness-of-fit results. The goodness of fit results showed that Model 3 (R^2^ = 17.5%) has a better predictive ability than the other models.

### Subgroup analyses by potential effect modifiers

**Figure 3:**
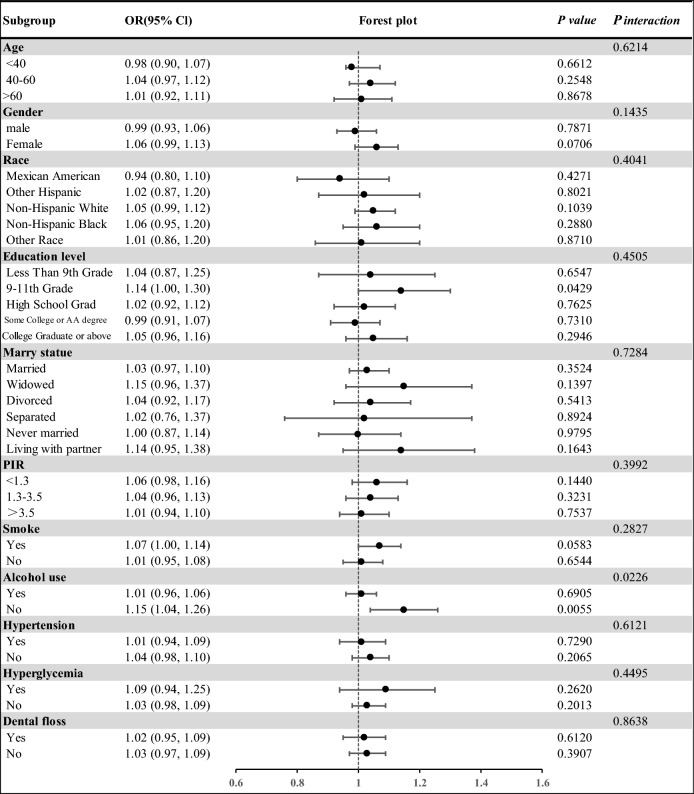
Subgroup analysis of the association between NHHR and periodontitis.

The findings of the subgroup analyses can be found in Fig. 3. The logistic regression analysis results for the subgroups showed statistically significant findings for participants with 9–11 grade education level or non-drinkers. Figure 3 displays that the OR of participants with 9–11 grade education level was 1.14 (1.00, 1.30), indicating that the prevalence of periodontitis increased by 14% for each unit increase in NHHR in this subgroup. However, the difference between the subgroups was not significant at 0.7284.

## Discussion

According to this study, NHHR serves as a satisfactory diagnostic biomarker for periodontitis. Our findings show a positive association between NHHR and an increased prevalence of periodontitis in an adjusted model. For each unit increase in NHHR, there is a 7% increase in the prevalence of periodontitis.

Metabolic abnormalities in lipid metabolism are common in patients with periodontitis and are associated with the occurrence and development of periodontitis^35,36^. NHHR is an emerging comprehensive indicator of atherosclerotic lipids, including HDL and NHDL, and is associated with dyslipidemia related diseases^28^. HDL are small lipoproteins that circulate throughout the body and are present near most cells^37^. In the mid-1950s, John Gofman and his colleagues discovered a negative correlation between levels of HDL cholesterol (HDL-C) and the likelihood of coronary heart disease^38^. Although it has been long acknowledged that high levels of HDL are inversely and linearly associated with the risk of systemic diseases like cardiovascular disease^39–41^, recent epidemiological studies have proposed that there is a “U-shaped” curve between HDL-C levels and cardiovascular disease risk, wherein patients with cardiovascular disease and excessively low or high levels of HDL-C have higher mortality rates compared to those with intermediate HDL-C levels^42–44^. Patients with low and high levels of HDL cholesterol have a higher mortality rate than those with intermediate levels, marking a significant deviation from earlier studies.

Over the past few years, there has been a growing interest in the connection between HDL and periodontitis. While certain studies have not detected disparities in serum HDL levels between healthy individuals and patients with periodontal disease^45–47^, two recent systematic reviews and meta-analyses have demonstrated a correlation between periodontitis and reduced serum HDL levels^20,21^. No clear “U-shaped” curve was found between NHHR and periodontitis in this study, potentially due to non-HDL-C’s influence on the relationship or the complex relationship between HDL levels and periodontitis, which may be mediated by multiple factors. Additional adjustment of relevant covariates is still necessary.

Further studies found that periodontal treatment significantly increased serum HDL-C levels in patients with periodontitis^16^. A bidirectional relationship between HDL and periodontitis has also been proposed: upregulation of proinflammatory factors caused by periodontitis can have adverse effects on serum lipid metabolism, as proposed by Fentoğlu et al.^48^ A significant correlation was identified between the pro-inflammatory cytokines TNF-α and IL-1b and the TC/HDL ratio in the gingival sulcus fluids and serum of hyperlipidemic patients with periodontitis. Furthermore, Anniina’s study validated these findings and proposed that HDL may play a role in the association between the number of teeth with deep periodontal pockets and C-reactive protein levels^49^.

Apart from HDL-C, NHDL is another crucial component of NHHR, capable of measuring low-density lipoprotein cholesterol (LDL-C), very-low-density lipoprotein cholesterol (VLDL-C), apolipoprotein A (apo A), and intermediate-density lipoprotein (IDL). Macri et al.^50^, based on their study involving experimental periodontitis in animals, found that a cholesterol-rich diet increases serum NHDL levels and exacerbates alveolar bone loss caused by periodontitis. Furthermore, various studies have reported higher levels of NHDL, including LDL-C, VLDL-C, and IDL, in patients with periodontitis compared to healthy individuals^51–54^. In a Mendelian randomization study published in 2023, it was also discovered through genetic predictions that apo A1 is associated with the risk of periodontitis^19^. The association between NHHR and periodontitis may be based on a joint analysis of the potential impact of two cholesterol categories on periodontitis, namely HDL and NHDL. However, this association and the exploration of mechanisms between them needs to be further explored with more well-designed basic and clinical studies based on large samples.

There were several limitations to this study: (1) Cohort studies are more reliable for validating the results of this study. The study was based on cross-sectional data, and the results may be influenced by selection bias; (2) Lipid profiles were assessed and recorded only once in this study. The lack of repeated measurements of lipid profiles may be subject to acute stress and incidental effects; (3) Some of the covariates have potential confounding power, are affected by the 2009–2014 NHANES database, there are problems with missing data, and there may be some bias in the results. Meanwhile, detailed medication information for patients, including types of medications, frequency, and duration, was not investigated in this study, and the impact of medication on periodontitis could not be ruled out.

## Conclusion

In summary, current research suggests a positive correlation between NHHR and the increased prevalence of periodontitis, potentially serving as a novel predictive factor for periodontal events. This also provides valuable evidence for primary prevention in individuals at high prevalence of periodontitis. Clinicians can use NHHR to assist in identifying high-risk populations for periodontitis, thereby improving screening efficiency.

## Data Availability

The data utilized and examined in this study are available upon reasonable request from the corresponding author.

## References

[1] Yang B, Pang X, Li Z (2021). Immunomodulation in the treatment of periodontitis: Progress and perspectives. Front. Immunol..

[2] Kwon T, Lamster IB, Levin L (2021). Current concepts in the management of periodontitis. Int. Dent. J..

[3] Lin H, Chen H, Zhao X (2022). Advances of exosomes in periodontitis treatment. J. Transl. Med..

[4] Spencer, L. J. *et al. *Global, regional, and national incidence, prevalence, and years lived with disability for 354 diseases and injuries for 195 countries and territories, 1990–2017: A systematic analysis for the Global Burden of Disease Study 2017. *Lancet*, **392** (10159), 1789–1858 (2018). 10.1016/S0140-6736(18)32279-7 PMC6227754 30496104

[5] Kassebaum NJ, Bernabé E, Dahiya M (2014). Global burden of severe periodontitis in 1990–2010: A systematic review and meta-regression. J. Dent. Res..

[6] Peres MA, Macpherson LMD, Weyant RJ (2019). Oral diseases: A global public health challenge. Lancet.

[7] Eke PI, Borgnakke WS, Genco RJ (2020). Recent epidemiologic trends in periodontitis in the USA. Periodontology 2000.

[8] Hajishengallis G (2022). Interconnection of periodontal disease and comorbidities: Evidence, mechanisms, and implications. Periodontology 2000.

[9] Genco RJ, Sanz M (2020). Clinical and public health implications of periodontal and systemic diseases: An overview. Periodontology 2000.

[10] Sulijaya B, Takahashi N, Yamazaki K (2019). Host modulation therapy using anti-inflammatory and antioxidant agents in periodontitis: A review to a clinical translation. Arch. Oral Biol..

[11] Balta MG, Papathanasiou E, Blix IJ (2021). Host modulation and treatment of periodontal disease. J. Dent. Res..

[12] Plemmenos G, Evangeliou E, Polizogopoulos N (2021). Central regulatory role of cytokines in periodontitis and targeting options. Curr. Med. Chem..

[13] Jørgensen HL, Lind BS (2022). Blood tests—Too much of a good thing. Scand. J. Prim. Health Care.

[14] Buss LF, Spitzer D, Watson JC (2023). Can I have blood tests to check everything is alright?. BMJ.

[15] Hu J, Xi D, Zhao J (2016). High-density lipoprotein and inflammation and its significance to atherosclerosis. Am. J. Med. Sci..

[16] Ehteshami A, Shirban F, Bagherniya M (2023). The association between high-density lipoproteins and periodontitis. Curr. Med. Chem..

[17] Zhu H, Ye G, Xie Y (2022). Association of high-density lipoprotein cholesterol and periodontitis severity in Chinese elderly: A cross-sectional study. Clin. Oral Investig..

[18] Koshy BS, Mahendra J (2017). The association between periodontal status, serum lipid levels, lipoprotein associated phosholipase A2 (Lp-PLA2) in chronic periodontitis subjects and healthy controls. J. Clin. Diagn. Res..

[19] Hu G, Song C, Yang Y (2023). Causal relationship between circulating lipid traits and periodontitis: Univariable and multivariable Mendelian randomization. Front. Endocrinol. (Lausanne).

[20] Xu J, Duan X (2020). Association between periodontitis and hyperlipidaemia: A systematic review and meta-analysis. Clin. Exp. Pharmacol. Physiol..

[21] Nepomuceno R, Pigossi SC, Finoti LS (2017). Serum lipid levels in patients with periodontal disease: A meta-analysis and meta-regression. J. Clin. Periodontol..

[22] Sheng G, Liu D, Kuang M (2022). Utility of non-high-density lipoprotein cholesterol to high-density lipoprotein cholesterol ratio in evaluating incident diabetes risk. Diabetes Metab. Syndr. Obes. Targets Ther..

[23] Kim SW, Jee JH, Kim HJ (2013). Non-HDL-cholesterol/HDL-cholesterol is a better predictor of metabolic syndrome and insulin resistance than apolipoprotein B/apolipoprotein A1. Int. J. Cardiol..

[24] Zuo PY, Chen XL, Liu YW (2015). Non-HDL-cholesterol to HDL-cholesterol ratio as an independent risk factor for the development of chronic kidney disease. Nutr. Metab. Cardiovasc. Dis..

[25] Iannuzzi A, Giallauria F, Gentile M (2021). Association between non-HDL-C/HDL-C ratio and carotid intima-media thickness in post-menopausal women. J. Clin. Med..

[26] Yang S, Zhong J, Ye M (2020). Association between the non-HDL-cholesterol to HDL-cholesterol ratio and non-alcoholic fatty liver disease in Chinese children and adolescents: A large single-center cross-sectional study. Lipids Health Dis..

[27] Lin W, Luo S, Li W (2023). Association between the non-HDL-cholesterol to HDL-cholesterol ratio and abdominal aortic aneurysm from a Chinese screening program. Lipids Health Dis..

[28] Sheng G, Liu D, Kuang M (2022). Utility of non-high-density lipoprotein cholesterol to high-density lipoprotein cholesterol ratio in evaluating incident diabetes risk. Diabetes Metab. Syndr. Obes..

[29] Qi X, Wang S, Huang Q (2024). The association between non-high-density lipoprotein cholesterol to high-density lipoprotein cholesterol ratio (NHHR) and risk of depression among US adults: A cross-sectional NHANES study. J. Affect. Disord..

[30] Du M, Mo Y, Li A (2023). Assessing the surveillance use of 2018 EFP/AAP classification of periodontitis: A validation study and clustering analysis. J. Periodontol..

[31] Li X, Wang L, Yang L (2024). The association between plain water intake and periodontitis in the population aged over 45: A cross-sectional study based on NHANES 2009–2014. BMC Oral Health.

[32] Chen X, Sun J, Zeng C (2024). Association between life’s essential 8 and periodontitis: A population-based study. BMC Oral Health.

[33] Weng J, Mao Y, Xie Q (2024). Gender differences in the association between healthy eating index-2015 and hypertension in the US population: Evidence from NHANES 1999–2018. BMC Public Health.

[34] Barros AJ, Hirakata VN (2003). Alternatives for logistic regression in cross-sectional studies: An empirical comparison of models that directly estimate the prevalence ratio. BMC Med. Res. Methodol..

[35] Morita T, Ogawa Y, Takada K (2009). Association between periodontal disease and metabolic syndrome. J. Public Health Dent..

[36] Sun J, Guo G (2023). Association between atherogenic index of plasma and periodontitis among U.S. adults. BMC Oral Health.

[37] von Eckardstein A, Nordestgaard BG, Remaley AT (2023). High-density lipoprotein revisited: Biological functions and clinical relevance. Eur. Heart J..

[38] Gofman JW, Glazier F, Tamplin A (1954). Lipoproteins, coronary heart disease, and atherosclerosis. Physiol. Rev..

[39] Pekkanen J, Linn S, Heiss G (1990). Ten-year mortality from cardiovascular disease in relation to cholesterol level among men with and without preexisting cardiovascular disease. N. Engl. J. Med..

[40] Després JP, Lemieux I, Dagenais GR (2000). HDL-cholesterol as a marker of coronary heart disease risk: The Québec cardiovascular study. Atherosclerosis.

[41] Jeppesen J, Hein HO, Suadicani P (1997). Relation of high TG-low HDL cholesterol and LDL cholesterol to the incidence of ischemic heart disease. An 8-year follow-up in the Copenhagen male study. Arterioscler. Thromb. Vasc. Biol..

[42] Ko DT, Alter DA, Guo H (2016). High-density lipoprotein cholesterol and cause-specific mortality in individuals without previous cardiovascular conditions: The CANHEART study. J. Am. Coll. Cardiol..

[43] Zvintzou E, Karampela DS, Vakka A (2021). High density lipoprotein in atherosclerosis and coronary heart disease: Where do we stand today?. Vasc. Pharmacol..

[44] Madsen CM, Varbo A, Nordestgaard BG (2017). Extreme high high-density lipoprotein cholesterol is paradoxically associated with high mortality in men and women: Two prospective cohort studies. Eur. Heart J..

[45] Sridhar R, Byakod G, Pudakalkatti P (2009). A study to evaluate the relationship between periodontitis, cardiovascular disease and serum lipid levels. Int. J. Dent. Hyg..

[46] Joseph R, Nath SG, Joseraj MG (2011). Elevated plasma homocysteine levels in chronic periodontitis: A hospital-based case-control study. J. Periodontol..

[47] Taleghani F, Shamaei M, Shamaei M (2010). Association between chronic periodontitis and serum lipid levels. Acta Med. Iran.

[48] Fentoğlu Ö, Köroğlu BK, Hiçyılmaz H (2011). Pro-inflammatory cytokine levels in association between periodontal disease and hyperlipidaemia. J. Clin. Periodontol..

[49] Haro A, Saxlin T, Suominen AL (2017). Serum lipids modify periodontal infection—Interleukin-6 association. J. Clin. Periodontol..

[50] Macri E, Lifshitz F, Ramos C (2014). Atherogenic cholesterol-rich diet and periodontal disease. Arch. Oral Biol..

[51] Penumarthy S, Penmetsa GS, Mannem S (2013). Assessment of serum levels of triglycerides, total cholesterol, high-density lipoprotein cholesterol, and low-density lipoprotein cholesterol in periodontitis patients. J. Indian Soc. Periodontol..

[52] Fentoğlu Ö, Tözüm Bulut M, Doğan B (2020). Is the relationship between periodontitis and hyperlipidemia mediated by lipoprotein-associated inflammatory mediators?. J. Periodontal Implant Sci..

[53] Thomas B, Prasad RB, Shetty S (2017). Comparative evaluation of the lipid profile in the serum of patients with type II diabetes mellitus and healthy individuals with periodontitis. Contemp. Clin. Dent..

[54] Griffiths R, Barbour S (2010). Lipoproteins and lipoprotein metabolism in periodontal disease. Clin. Lipidol..

